# A brief exposure to cadmium impairs Leydig cell regeneration in the adult rat testis

**DOI:** 10.1038/s41598-017-06870-0

**Published:** 2017-07-24

**Authors:** Xiaolong Wu, Xiaoling Guo, Huang Wang, Songyi Zhou, Lili Li, Xiaomin Chen, Guimin Wang, Jianpeng Liu, Hong-Shan Ge, Ren-Shan Ge

**Affiliations:** 1 Department of Anesthesiology, The Second Affiliated Hospital and Yuying Children’s Hospital of Wenzhou Medical University, 109 Xueyuan West Road, Wenzhou, Zhejiang 325027 China; 2 Center of Scientific Research, The Second Affiliated Hospital and Yuying Children’s Hospital, Wenzhou Medical University, 109 Xueyuan West Road, Wenzhou, Zhejiang 325027 China

## Abstract

Cadmium is an endocrine disruptor, impairing male reproduction. The objective of this study is to investigate whether cadmium affects rat Leydig cell regeneration and to dissect the underlying mechanism. Adult male Sprague-Dawley rats received a single intraperitoneal injection (i.p.) of 0, 0.5 or 1.0 mg/kg of cadmium chloride, followed by ethane dimethane sulfonate (EDS) treatment to eliminate adult Leydig cells 20 days later. Compared to control (0 dose), cadmium treatment reduced serum testosterone levels by days 21, 35, and 56 after EDS treatment. Serum luteinizing hormone (LH) levels were also affected by day 56, the only time point examined. There were fewer regenerated Leydig cells in the cadmium-treated testis on days 35 and 56 after EDS treatment. Further studies demonstrated that the mRNA or protein levels of Leydig (*Lhcgr*, *Scarb1*, *Star*, *Cyp11a1*, *Hsd3b1*, *Cyp17a1*, *Hsd17b3*, and *Hsd11b1*), non-Leydig (*Fshr* and *Dhh*), and gonadotroph (*Lhb*) cells were also significantly lower in cadmium-treated animals. Since LH and desert hedgehog (DHH) are critical factors for Leydig cell differentiation, our result demonstrated that the lower doses of cadmium exposure, even briefly, may permanently damage Leydig cell regeneration.

## Introduction

In recent years, the increased incidence of human male infertility has been of a great concern. This is possibly related to the widespread usage of endocrine disrupting substances, including pesticides, food additives, plasticizers and industrial chemicals. Cadmium, a heavy metal, may also function as an endocrine disruptor, by interfering with the endocrine cell functions or their development. Human may expose to this metal by multiple dynamics. Cadmium has been used as the stabilizer in the manufacture of polyvinyl chloride polymer, preservative pigments, phosphate fertilizers, nuclear neutron filters, and batteries. Smoking is also a major source of cadmium intake. Each cigarette contains 5 mg of cadmium, which could lead to significantly higher levels of blood cadmium, with smokers of 4–5 times higher than that of non-smokers^1^. Different from other environmental contaminants, cadmium has an especially long elimination half-life of 20–40 years, leading to a significant accumulation in the human body. This makes the metal particularly dangerous and challenging.

Studies have showed that the mammalian testis is a sensitive target of cadmium toxicity^1–3^. Exposure to cadmium induced germ cell loss and decreased sperm production^4^, which may account for the increased incidence of male infertility. However, it seems that the testicular somatic cells such as Sertoli and Leydig cells are the targets of cadmium. Cadmium at low concentrations was capable of significantly affecting the morphology and functions of rat Sertoli cells^5^. Cadmium has also been shown to inhibit testosterone production in rodent Leydig cells^6^, possibly via interfering with the mitochondrial function^7^. Testosterone, produced by Leydig cells, is necessary for spermatogenesis^8^. Testosterone synthesis in Leydig cells involves with complex signaling pathway, steroid-transportation network, and biosynthetic enzyme functions (see review ref. 9). For example, in rats, luteinizing hormone (LH) secreted by the gonadotroph in the pituitary binds to its receptor, luteinizing hormone receptor (LHCGR, encoded by *Lhcgr*) in the Leydig cells (see review ref. 9), leading to a series of signaling cascade to increase the extracellular cholesterol intake via cell membrane scavenger receptor class B type 1 (SCARB1, encoded by *Scarb1*). The intracellular cholesterol is further mobilized and transported across the mitochondrial membrane by steroidogenic acute regulatory protein (STAR, encoded by *Star*) to the mitochondrial inner membrane, where the cholesterol side chain cleavage enzyme (CYP11A1, encoded by *Cyp11a1*) is located and catalyzes this substrate into pregnenolone. Pregnenolone diffuses from the mitochondria into the surrounding smooth endoplasmic reticulum, where by the functions of a series of steroidogenic enzymes, including 3β-hydroxysteroid dehydrogenase 1 (3β-HSD1, encoded by *Hsd3b1*), cytochrome P450 17α-hydroxylase/17,20-lyase (CYP17A1, encoded by *Cyp17a1*), and 17β-hydroxysteroid dehydrogenase 3 (17β-HSD3, encoded by *Hsd17b3*), this steroid is converted to the final product, testosterone.

In addition to affecting the function of adult Leydig cells, cadmium may also capable of interfering with the pubertal development of Leydig cells. Leydig cell development has been divided into four stages in the rat: stem, progenitor, immature, and adult Leydig cells^9^. The pubertal development of Leydig cells can also be mimicked by the regeneration of Leydig cells after a single intraperitoneal injection of 75 mg/kg ethane dimethane sulfonate (EDS) to the rat. On day 7 after EDS treatment, adult Leydig cells were completely eliminated^10^. On days 21, 35, and 56 after EDS treatment, Leydig cell regeneration progressed through progenitor, immature, and adult stages, as visualized by the staining of 3β-HSD1 (Leydig cell lineage biomarker) and 11β-hydroxysteroid dehydrogenase 1 (11β-HSD1, the immature and adult Leydig cell biomarker)^10^. The development or regeneration of Leydig cells are regulated by many factors, including gonadotroph-secreted LH and non-Leydig testicular cell factors (such as desert hedgehog, DHH, and anti-Müllerian hormone, AMH)^11, 12^. Both the expressions and secretions of DHH and AMH are regulated by the gonadotroph-secreted follicle-stimulating hormone (FSH, encoded by *Fshb*), which binds to its receptor (FSHR, encoded by *Fshr*) on Sertoli cells^13, 14^.

In the represent study, we investigated whether a brief exposure to lower doses of cadmium might affect Leydig cell regeneration in the adult rat testis and whether such effect, if arise, was caused by changes in the precursor cells or micro-environment that regulates the developmental cells.

## Materials and Methods

### Materials and Animals

Cadmium chloride and dimethyl sulfoxide were purchased from Sigma-Aldrich (St. Louis, MO). Smoothened agonist (SAG), an agonist of DHH, and EDS were purchased from Pterosaur Biotech Co. (Hangzhou, China). The manufacturers of antibodies were listed in Supplementary Table 1. Sixty 51-day-old male Sprague-Dawley rats were purchased from Laboratory Animal Center of Wenzhou Medical University (Wenzhou, China). All animal studies were approved by the Wenzhou Medical University’s Animal Care and Use Committee and were performed in accordance with the Guide for the Care and Use of Laboratory Animals.

### Animal Treatments

Rats were maintained in a 12 h dark/light cycle under temperature at 23 ± 2 °C, and relative humidity of 45% to 55%, with ad libitum access to food and water. Animals were adjusted for one week before they were randomly divided into 3 groups (20 animals per group). Cadmium chloride was dissolved in normal saline and was intraperitoneally injected (0.5 ml). Rats in group 1 received normal saline injection, serving as the control, while rats in groups 2 and 3 received a single i.p. of 0.5 or 1.0 mg/kg of cadmium chloride, respectively. Twenty days after treatment, rats in all groups received a single i.p. of 75 mg/kg EDS to eliminate Leydig cells. EDS was dissolved in a mixture of dimethyl sulfoxide and deionized sterile water (1:3, v/v), and was given by i.p. according to the previous study^10^. Rats (5 animals each group) were sacrificed on days 7, 21, 35, and 56 after EDS, by asphyxiation with CO_2_. Trunk blood was collected in a gel glass tube, and centrifuged at 1500× g for 10 min to collect serum. Serum samples were stored at −80 °C until analysis for LH and FSH by Elisa as well as testosterone by radioimmunoassay (RIA). Furthermore, each pair of testes was separated and weighted. One testis per animal was frozen in the liquid nitrogen and stored in −80 °C for subsequent analysis of gene and protein expression levels. The contralateral testis was punched using a needle and then fixed in Bouin’s solution for histochemical analysis. Meanwhile, pituitary was also collected and stored at −80 °C until extraction of total RNAs.

### Real-time polymerase chain reaction (qPCR)

Total RNAs were isolated from the testes and pituitaries using Trizol according to the manufacturer’s instructions (Invitrogen, Carlsbad, CA), and the concentration of RNA was quantified by measuring OD at 260 nm. The first strand of cDNA was synthesized and used for qPCR as previously described^15^. The expression levels of Leydig (*Lhcgr*, *Scarb1*, *Star*, *Cyp11a1*, *Hsd3b1*, *Cyp17a1*, *Hsd17b3*, and *Hsd11b1*), non-Leydig (*Fshr* and *Dhh*) and gonadotroph cell genes (*Lhb*, *Fshb*, and *Gnrhr*) were analyzed using a SYBR Green qPCR Kit (Takara, Otsu, Japan). The reaction mixture consisted of 10 μl SYBR Green Mix, 0.8 μl forward, 0.8 μl reverse primers, 1 μg diluted cDNA and 5–8 μl H_2_O. The reaction process was as follows: 95 °C for 5 min, followed by 40 cycles of 95 °C for 10 sec, and 60 °C for 30 sec. The relative expression of genes was normalized to β-Actin (*Actb*). The melting curve was examined for the quality of PCR amplification for each sample, and quantification was performed with the standard curve method. The primers were listed in Supplementary Table 2.

### Western blotting

Testes were homogenized and then were lysed with radio immunoprecipitation assay (RIPA) buffer (Bocai Biotechnology, China) to obtain total proteins. The protein concentrations of samples were measured with BCA^TM^ Protein Assay Kit (Takara, Japan) according to the manufacturer’s instruction. Aliquot of 50 μg of proteins were electrophoresed on 10% polyacrylamide gels containing sodium dodecyl sulfate and transferred onto nitrocellulose membranes. The membranes were blocked with 5% non-fat milk in Tris-buffered saline tween-20 buffer for 1 h. Then, the membranes were incubated overnight at 4 °C with primary antibodies against the following antigens: LHCGR, STAR, CYP11A1, 3β-HSD1, CYP17A1, 11β-HSD1, FSHR, DHH, and ACTB (listed in Supplementary Table 1). The membranes were then washed and incubated with HRP-conjugated anti-rabbit or anti-goat IgG secondary antibody (1:5000, Bioword, USA) for 1 h at room temperature and washed 3 times. The immunoreactive bands were visualized by chemiluminescence using a kit (ECL, Amersham, Arlington Heights, IL). The intensity of band was analyzed with ImageJ software.

### Tissue Array Preparation and Immunohistochemistry

One testis from each rat was used for immunohistochemical staining (Vectastain, Elite, ABC kit, PK-6101; Vector Laboratories, Inc., Burlingame, CA) according to the manufacturer’s instructions. Five testes per group at each time point were used and testis samples were prepared and then embedded in paraffin as a tissue array as below. Four micrometer-thick transverse sections were cut and mounted on the glass slides. Avidin-biotin immunostaining was conducted following manufacturer’s instructions (Vector, Burlingame, CA, USA). Antigen retrieval was performed by microwave irradiation for 10 min in 10 mM (pH 6.0) of citrate buffer, after which endogenous peroxidase was blocked with 0.5% of H_2_O_2_ in methanol for 30 min. Sections were then incubated with an 3β-HSD1 or 11β-HSD1 rabbit polyclonal antibody diluted 1:1000 for 1 h at room temperature. Diaminobenzidine was used for visualizing the antibody-antigen complexes, positively labeling Leydig cells by brown cytoplasmic staining. Mayer hematoxylin was applied as the counterstaining. The sections were then dehydrated in graded concentrations of alcohol and cover-slipped with resin (Permount, SP15-100; Fisher Scientific, Thermo Fisher Scientific, Waltham, UK). Non-immune rabbit IgG (Sigma-Aldrich, St. Louis, MO) was used in the incubation of the negative control sections at the working dilution the same as the primary antibody. The cells with positive stainings of 3β-HSD1 (all Leydig cells) or 11β-HSD1 (immature and adult Leydig cells) were counted. Leydig cell numbers were calculated using a stereological method as below.

### Enumeration of Leydig Cell Number

To enumerate 3β-HSD1 positive Leydig cell numbers or 11β-HSD1 positive immature and adult Leydig cell numbers, sampling of the testis was performed according to a fractionator technique as previously described^16^. In brief, five testes per group at each time point were used. Each testis was cut in 8 discs and two discs were randomly selected. Then discs were cut in 4 pieces and one piece was randomly selected from total 8 pieces. These pieces of testis were embedded in paraffin in a tissue array. Paraffin blocks were sectioned in 4 μm-thick sections. About ten sections were randomly sampled from each testis per rat. Sections were used for immunohistochemical staining. Identification of all cells in the Leydig cell lineage was performed by the staining with a polyclonal antibody specific for the 3β-HSD1, and identification of immature and adult Leydig cells was performed by the staining with a polyclonal antibody specific for the 11β-HSD1 as above because this biomarker is present in Leydig cells at the advanced stages^17^. Using the live image of a digital camera, under a 10× objective, and starting at a fixed point of the “upper” sections, total microscopic fields per section were counted. The total number of Leydig cells was calculated by multiplying the number of Leydig cells counted in a known fraction of the testis by the inverse of the sampling probability.

### Seminiferous Tubule Isolation and Culture

The seminiferous tubule isolation and culture was performed as previously described^18^. One 56-day-old male Sprague Dawley rat was i.p. injected with a single dose of EDS (75 mg/kg body weight). The rat was sacrificed by decapitation four days after EDS treatment, when all Leydig cells were eliminated^19, 20^. Testes were placed in MEM-199 culture medium and decapsulated. Seminiferous tubules were mechanically separated from the interstitium^18^. The tubules were distributed randomly into 12-well plates, with each well containing tubule fragments of the equivalent total length (about 1 cm). Tubules were cultured at 34 °C and 5% CO_2_ for up to 4 weeks in a 1:1 mixture of DMEM/F-12 and Medium 199 (pH 7.2), sodium bicarbonate (2.2 mg/ml), bovine serum albumin (1 mg/ml), and penicillin/streptomycin (100 U/ml and 100 µg/ml, respectively). The medium containing insulin-transferrin-sodium selenite was served as the control. Media containing LH (10 ng/ml), or SAG (0.5 μM), or SAG (0.5 μM) + LH (10 ng/ml) were used as the treatment groups. Triplicate wells were used at each time point for 21 days, and each experiment was repeated at least three times. Media were collected and frozen (−20 °C) for testosterone measurement, and tubules were immediately fixed for the morphological study.

### Serum and Medium Testosterone Assay

Serum and medium concentrations of testosterone were measured by the tritium based radioimmunoassay, as previously described^21^. Standards ranging between 10 and 2000 pg/ml testosterone were prepared in triplicate. Standards and samples were incubated with respective tracer and antibody at 4 °C overnight and charcoal-dextran suspension was used to separate the bound and free steroids. The bound steroids were mixed with a scintillation buffer and counted in a β-scintillation counter (PE, USA). The minimum detectable concentration of the assay for testosterone was 5 pg/ml. Internal control contains 100 pg/ml testosterone dissolved in the same culture media. Interassay and intraassay coefficients of variation for testosterone were less than 15%.

### ELISA for Serum LH and FSH

The serum levels of LH and FSH were detected with enzyme linked immunosorbent assay (ELISA) kit according to the manufacturer’s instructions (Chemicon CA, USA). Briefly, 200 μl samples and 50 μl assay diluent were added to pre-coated 96-well plates. The plates were incubated for 2 h at room temperature, and washed 5 times with washing buffer. 100 μl peroxidase-conjugated IgG anti-LH or anti-FSH of solution was added into each well for 2 h at room temperature. Then plates were washed 5 times. Then 100 μl substrate buffers were added into each well, and incubated in the dark place for 30 min at room temperature. The enzyme reaction was stopped by 50 μl stop solution. The quantification of LH and FSH levels were obtained by a microplate reader at 550 nm with correction wavelength at 450 nm. Data was analyzed by GraphPad Prism software.

### Statistical analysis

All data are presented as the mean ± standard errors (SE). Statistical significance was analyzed using one-way ANOVA with post hoc Dunnett’s multiple comparisons test to the control. Statistical analyses were performed using GraphPad Prism (version 6, GraphPad Software Inc., San Diego, CA, USA). P < 0.05, 0.01 or 0.001 were considered statistically significant.

## Results

### General Reproductive Toxicology

Intraperitoneal injection of rats with 0.5 and 1.0 mg/kg cadmium caused a decreased body weight growth rate compared with the control (Table 1). Cadmium also caused a dose-dependent decrease of testis weight. These results are in consistent with those observed in a previous study^2^.

**Table 1:** General parameters of toxicology after treatment of CdCl and EDS

Parameters	Dosage (mg/kg)		
	0	0.5	1.0
**Body weight**			
Before-EDS	305.5 ± 15.6	307.8 ± 16.25	304.11 ± 15.05
Post-EDS day 0	421.75 ± 25.52	393.64 ± 3.5**	378 ± 23.279**
Post-EDS day 21	438.57 ± 31.94	398.4 ± 30.17*	386.73 ± 37.54**
Post-EDS day 35	459.8 ± 13.90	413.3 ± 34.53**	398.8 ± 10.9**
Post-EDS day 56	519.6 ± 19.4	486.2 ± 19.94*	466 ± 15.5**
**Testes weight**			
7 days post-EDS	1.54 ± 0.045	1.38 ± 0.043**	1.29 ± 0.019**
21 days post-EDS	1.79 ± 0.047	1.55 ± 0.036**	1.45 ± 0.06**
35 days post-EDS	1.95 ± 0.046	1.78 ± 0.046**	1.63 ± 0.079**
56 days post-EDS	2.15 ± 0.08	1.90 ± 0.068**	1.77 ± 0.078**

Mean ± SE, n = 5. *P < 0.05, **P < 0.01 indicate significant difference when compared to control (0 mg/kg) at each time point.

### Cadmium Reduces Serum Testosterone levels

**Figure 1:**
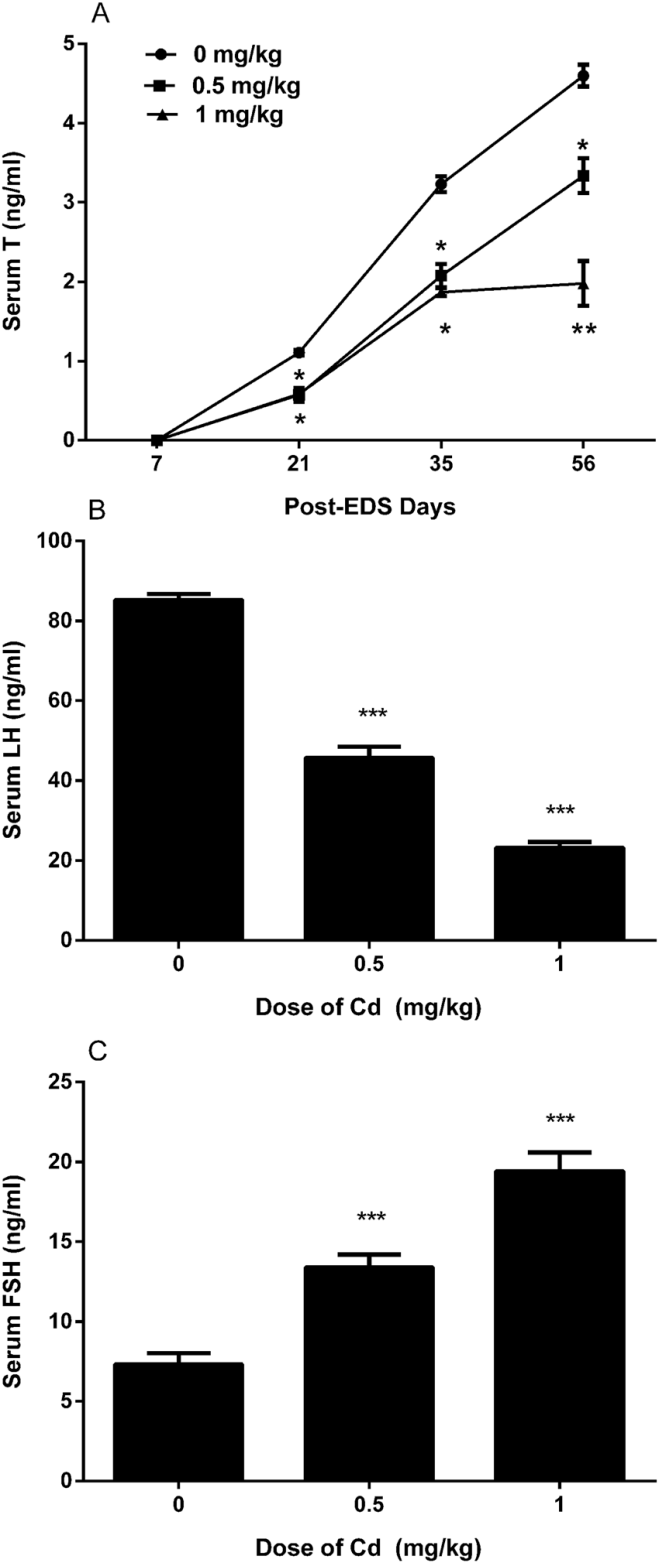
The levels of serum testosterone (T) on days 7, 21, 35, and 56 as well as serum LH and FSH levels on day 56 after EDS treatment with or without cadmium administration. Panel A, testosterone; Panel B, LH; and Panel C, FSH. Mean ± SE, n = 5. *P < 0.05, **P < 0.01, and ***P < 0.001 indicate significant differences when compared to control (0 mg/kg) at each time point.

In all groups, on day 7 after treatment of EDS, serum testosterone levels were undetectable, indicating that Leydig cells were completely eliminated. Testosterone levels in all groups were gradually elevated starting on post-EDS day 21. Cadmium (0.5 or 1.0 mg/kg) showed a significant decrease of testosterone values compared with the control at the same time point (Fig. 1A). Results suggest that Leydig cell regeneration could be retarded by cadmium.

### Cadmium Reduces Serum LH but Increases FSH levels

Serum LH and FSH levels were measured in all samples on day 56 after EDS treatment. Serum LH levels (Fig. 1B) showed significant decreases while serum FSH levels (Fig. 1C) showed significant increases after cadmium exposure.

### Cadmium Down-regulates Leydig and Sertoli Cell Genes

**Figure 2:**
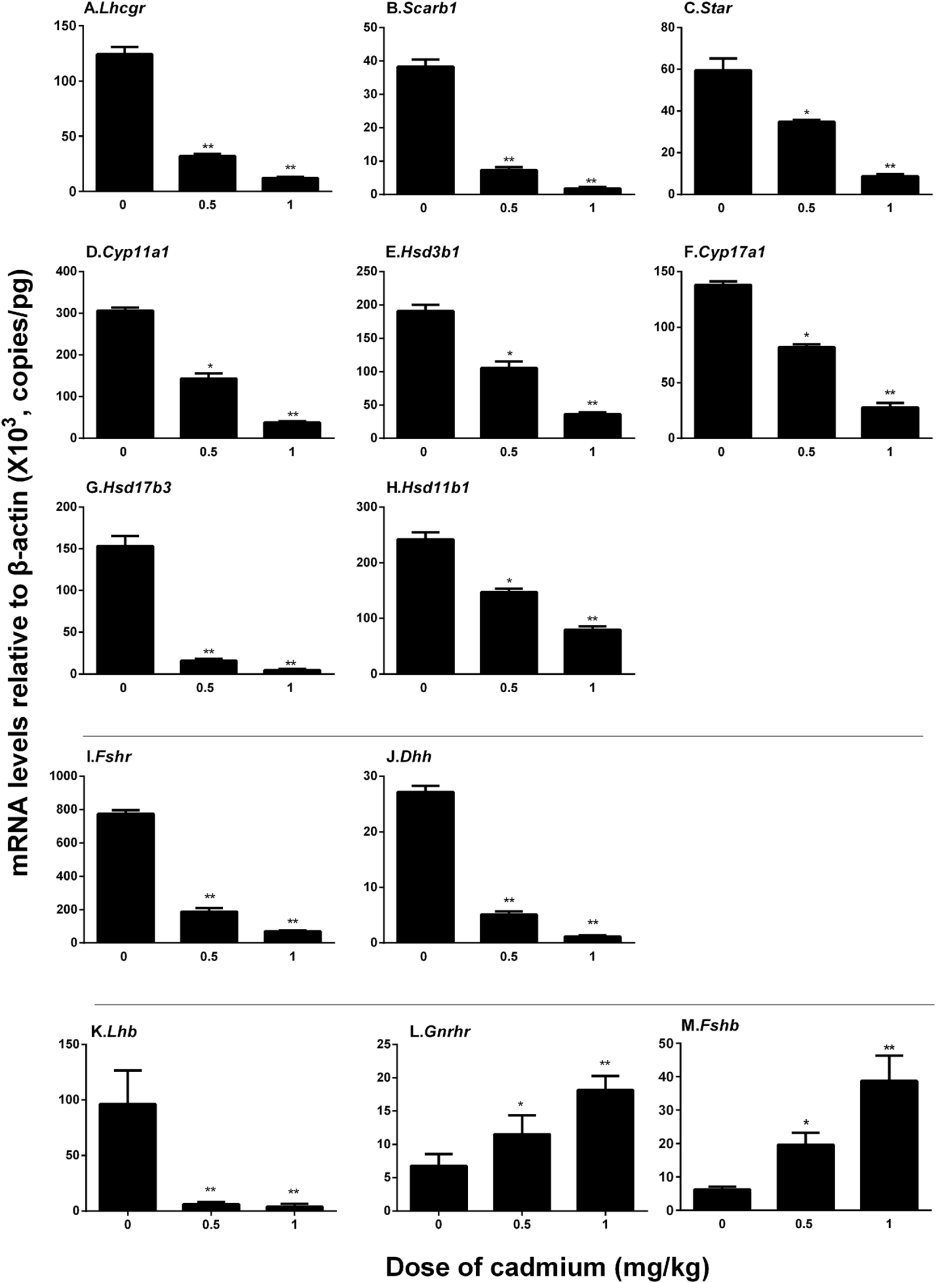
Gene expression levels of the testis and the pituitary on post-EDS day 56. Leydig cell genes: (**A**) *Lhcgr*, (**B**) *Scarb1*, (**C**) *Star*, (**D**) *Cyp11a1*, (**E**) *Hsd3b1*, (**F**) *Cyp17a1*, (**G**) *Hsd17b1*, (**H**) *Hsd11b1*, and (**I**) *Amh*; non-Leydig cell genes: (**J**) *Fshr* and (**K**) *Dhh*; Gonadotroph genes: (**L**) *Lhb*, (**M**) *Gnrhr*, and (**N**) *Fshb*. Mean ± SE, n = 5. *P < 0.05, **P < 0.01 indicate significant differences when compared to control (0 mg/kg).

We measured the expression levels of Leydig cell specific genes (*Lhcgr*, *Scarb1*, *Star*, *Cyp11a1*, *Hsd3b1*, *Cyp17a1*, *Hsd17b3*, and *Hsd11b1*) and non-Leydig cell genes (*Fshr* and *Dhh*) on post-EDS day 56 (Fig. 2). Cadmium dose-dependently decreased the levels of all these genes in the testis, indicating that cadmium impairs both Leydig and non-Leydig cell functions. We all also measured the expression levels of gonadotroph specific genes (*Lhb*, *Gnrhr*, and *Fshb*). *Lhb* and *Fshb* represent the rate-limiting bioavailable serum LH and FSH, respectively^22^. *Lhb* level was significantly down-regulated, conforming the serum LH levels. This indicates that a brief exposure to cadmium also disrupts pituitary LH secretion (Fig. 1B). Interestingly, *Gnrhr* and *Fshb* levels were elevated, indicating that the Sertoli cell function was disrupted and which may reduce the negative feedback regulations in the pituitary (Fig. 1C).

### Cadmium Reduces Leydig and non-Leydig Cell Protein Levels

**Figure 3:**
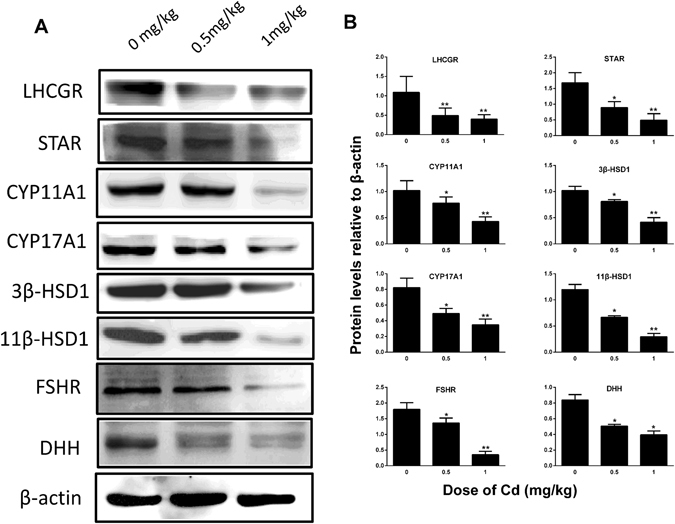
Protein levels of Leydig and Sertoli cells. Leydig cell proteins: (**A**) Western blot band. (**B**) Quantification of protein levels. Mean ± SE, n = 5. *P < 0.05, **P < 0.01 indicate significant differences when compared to control (0 mg/kg).

We measured the levels of Leydig cell proteins (LHCGR, STAR, CYP11A1, CYP17A1, 3β-HSD1, and 11β-HSD1) and non-Leydig cell proteins (FSHR and DHH) in the testes on post-EDS day 56 (Fig. 3A). As shown in Fig. 3B, the quantification results showed that cadmium dose-dependently lowered the levels of all these proteins which were in parallel with those of their respective mRNA levels. Our results further confirmed that cadmium impaired the Leydig and non-Leydig cell functions.

### Cadmium Reduces Leydig Cell Number

**Figure 4:**
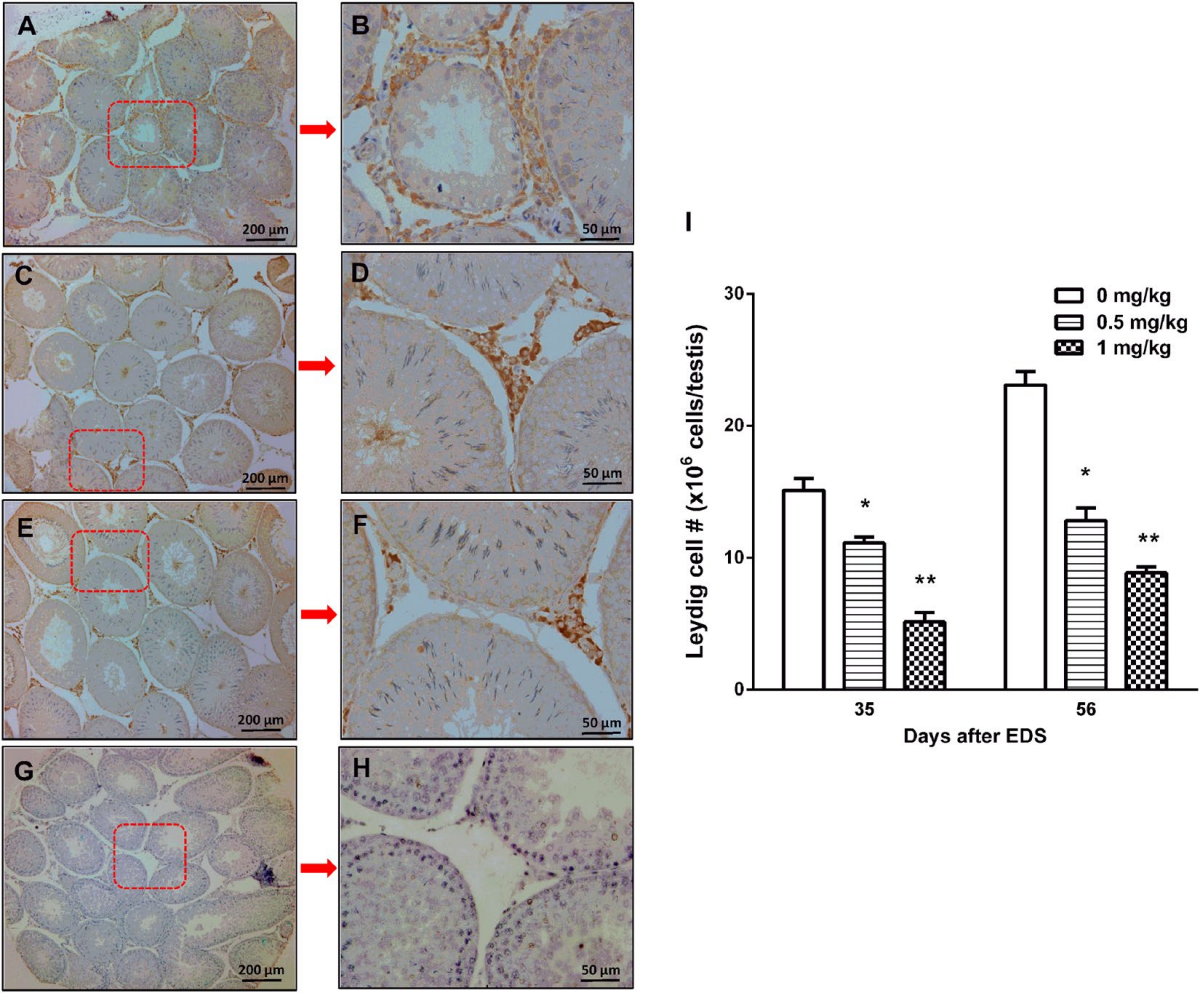
Immunohistochemical staining of 3β-hydroxysteroid dehydrogenase (3β-HSD1) in rat testis sections on days 35 and 56 after EDS treatment. (**A**) and (**B**) for control group on days 35 and 56; (**C**) and (**D**) for 0.5 mg/kg CdCl on days 35 and 56; (**E**) and (**F**) for 1.0 mg/kg CdCl on days 35 and 56; (**G**) and (**H**) for the negative control. (**I**) Quantification of 3β-HSD1 positive Leydig cell numbers on days 35 and 56. Mean ± SE, n = 5. *P < 0.05, **P < 0.01 indicate significant difference when compared to control (CON) at each time point.

**Figure 5:**
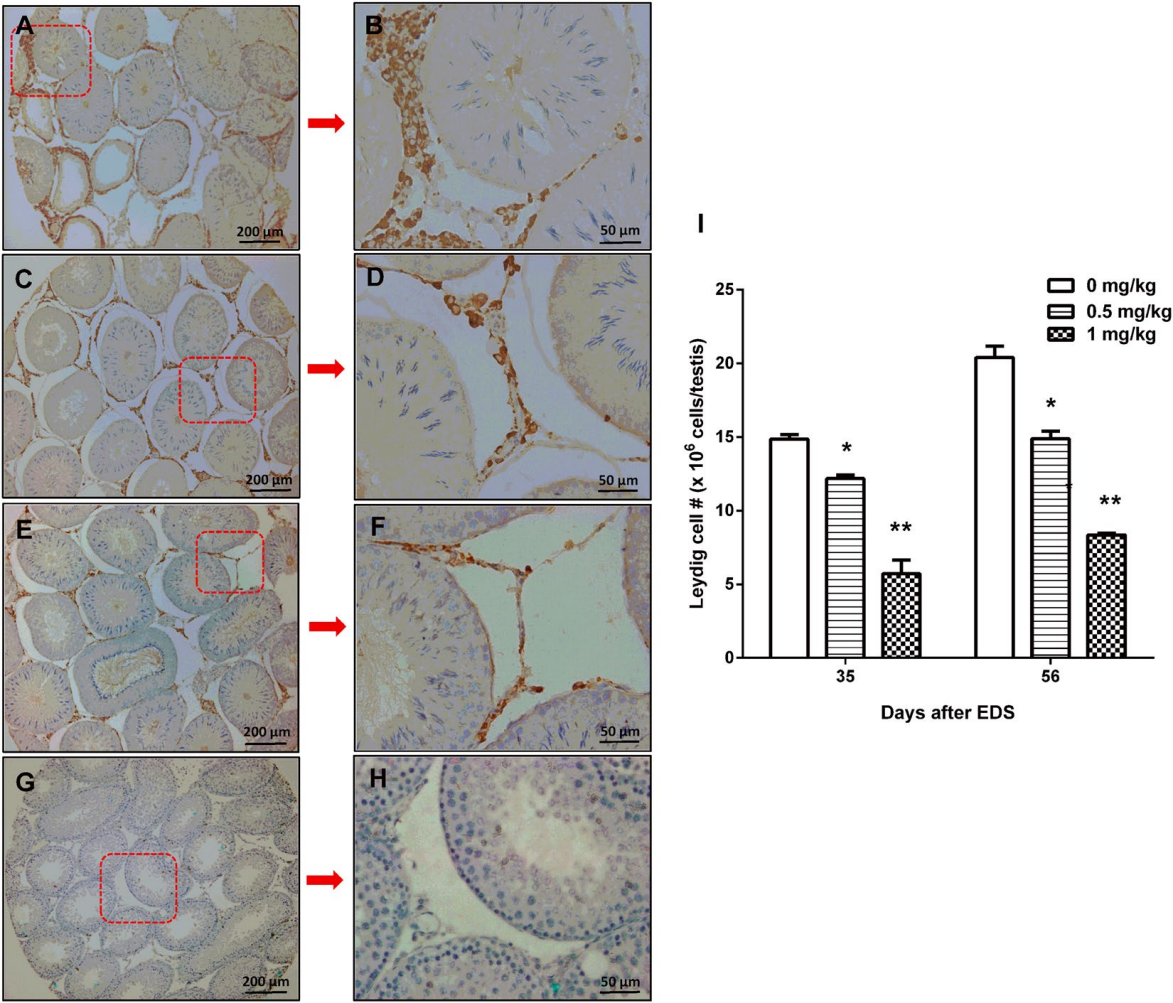
Immunohistochemical staining of 11β-hydroxysteroid dehydrogenase (11β-HSD1) in rat testis sections on days 35 and 56 after EDS treatment. (**A**) and (**B**) for control on days 35 and 56; (**C**) and (**D**) for 0.5 mg/kg CdCl on days 35 and 56; (**E**) and (**F**) for 1.0 mg/kg CdCl on days 35 and 56; (**G**) and (**H**) for the negative control. (**I**) Quantification of 11β-HSD1 positive Leydig cell numbers on days 35 and 56. Mean ± SE, n = 5. *P < 0.05, **P < 0.01 indicate significant difference when compared to control (CON) at each time point.

Reductions in testosterone productions and steroidogenic protein/mRNA levels may due to either fewer number of Leydig cells formed or lower quality of Leydig cells formed or both. To further analyze whether cadmium exposure affects the progress and the number of Leydig cells generated, we stained Leydig cells with its biomarker 3β-HSD1 on 35 and 56 days after EDS treatment. When compared to control, cadmium dose-dependently decreased 3β-HSD1 positive Leydig cell number (Fig. 4), indicating that cadmium impairs the Leydig cell regeneration process. We further stained Leydig cells with 11β-HSD1, which detects Leydig cells in the advanced stages. When compared to control, cadmium dose-dependently decreased 11β-HSD1-postive Leydig cell number (Fig. 5). However, we did not observe the difference between numbers of 3β-HSD1 and 11β-HSD1 positive cells. These indicate that the cadmium reduces the number of Leydig cells possibly before 35 days after EDS, a stage for stem Leydig cells into immature Leydig cells.

### DHH and LH Are Essential for Stem Leydig Cell Differentiation

**Figure 6:**
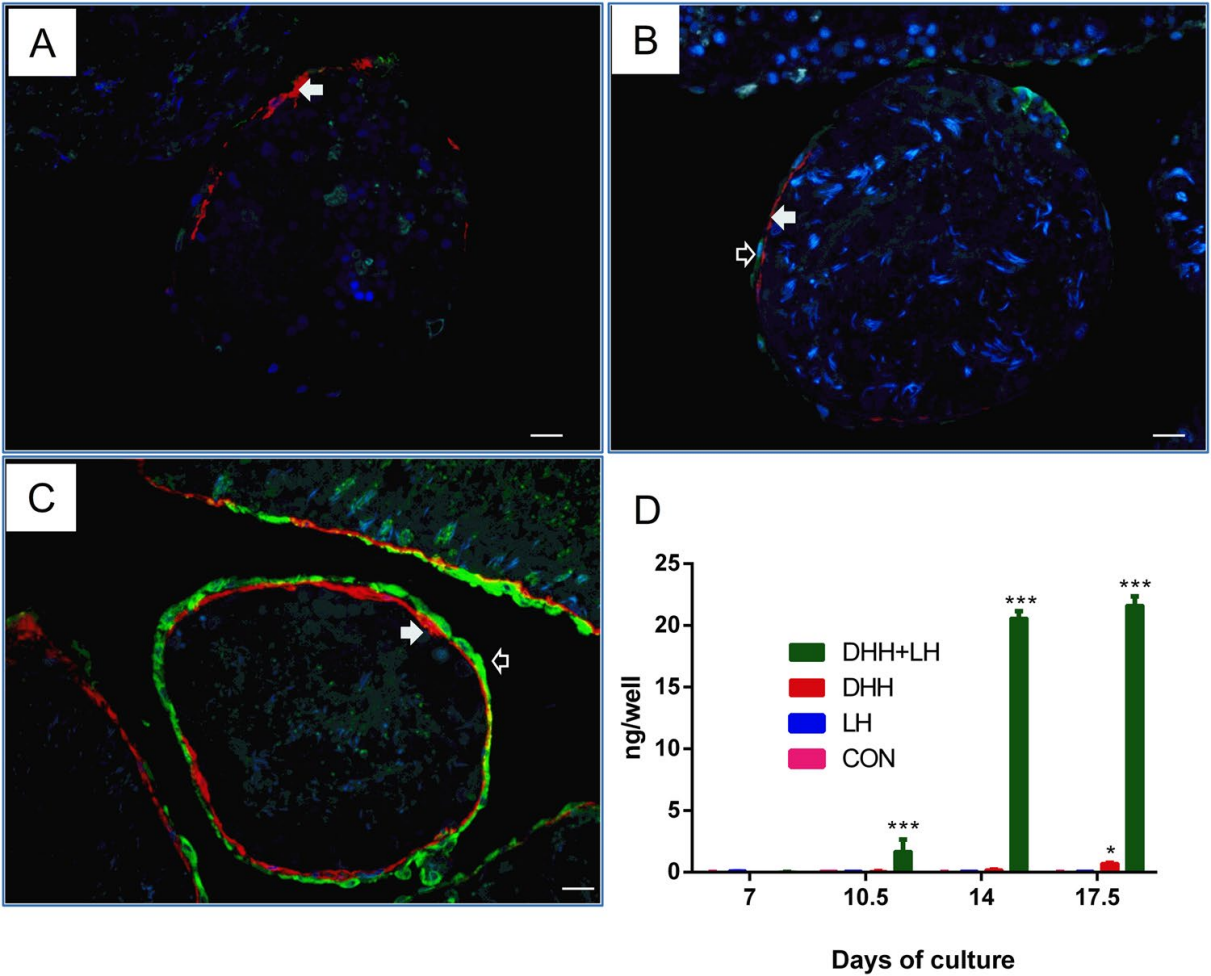
Differentiation of Leydig cells using an *in vitro* culture system of rat seminiferous tubules. 11β-HSD1 staining (green color, unfilled arrow) showed the formation of Leydig cells at the advanced stages. α-Smooth muscle actin staining (red color, solid arrow) showed the myoid cells, which circle the seminiferous tubules. 11β-HSD1 positive cells are outside the α-smooth muscle actin positive cells, indicating that they were differentiated from the stem Leydig cells on the surface of the tubule. Immunohistochemical staining of tubules after 21 days of culture: Panel A, with LH alone; panel B with DHH alone; and panel C with DHH and LH. Medium testosterone (T) levels during the course of culture (panel D). Mean ± SE, n = 6. *P < 0.05, ***P < 0.001 indicate significant differences when compared to control (CON) at each time point.

Since we found that there was significant reduction of DHH and LH expressions, we would find that whether DHH and LH are essential for stem Leydig cell differentiation. We setup an *in vitro* stem Leydig cell differentiation model using the culture of EDS-treated rat seminiferous tubules. As shown in Fig. 6A, after culture for 21 days, there were almost no Leydig cells present on the surface of the seminiferous tubule in the control medium (only LH). When cultured with DHH alone, there were just some Leydig cells which were differentiated (Fig. 6B, green-color with 11β-HSD1 staining). When LH and DHH in combination, many Leydig cells were formed (Fig. 6C). The medium testosterone was significantly and robustly increased by DHH and LH starting on day 14 (Fig. 6D). This further confirmed that DHH and LH combined were very critical for the differentiation of stem Leydig cells and that the decreased expression levels of DHH in the testis and LH in the pituitary might well be the reasons that Leydig cell development is delayed in cadmium-exposed animals.

## Discussion

In this study, we took advantage of the EDS-induced rat Leydig cell regeneration model, which is very unique to study Leydig cell developmental process in the adult rat testis. Adult population of Leydig cells in the testis are completely eliminated by a single intraperitoneal injection of 75 mg/kg EDS^23^. The loss of Leydig cells results in the elevation of circulating luteinizing hormone (LH) and increased secretion of local factors, two necessary changes that enable the regeneration process to take place^23, 24^. Similar to the developmental process during the puberty, Leydig cell regeneration in adult testis also undergoes 3 stages: progression of stem Leydig cells into progenitor by day 21, and then into immature cells by day 35, and finally into adult Leydig cells by day 56 after EDS treatment^10^. In the present study, we used this model system to test whether environmental contaminant cadmium affects Leydig cell development.

A single low dose exposure of cadmium seemed affecting Leydig cell development significantly. First, cadmium exposure, at both doses, decreased serum testosterone levels following EDS treatment. Second, the expression levels of Leydig cell steroidogenic genes, and their protein products, were all reduced in cadmium treated animals. These include *Lhcgr*, *Scarb1*, *Star*, *Cyp11a1*, *Hsd3b1*, *Cyp17a1*, *Hsd17b3* and *Hsd11b1*. Since these analyses were done based on the whole testis, we further examined whether cadmium exposure affected Leydig cell numbers. Expression of 3β-HSD1 was considered to be a marker for the identification of all developmental stages of Leydig cells since the cells begin to express this protein as soon as they entered Leydig lineage (progenitor stage), while expression of 11β-HSD1 were considered to be only happened at the late stages of the development (immature and adult stages). Quantification of Leydig cells based on these two markers consistently indicated that cadmium treatment significantly reduced the number of Leydig cells generated after EDS treatment, although we did not observe the difference between numbers of 3β-HSD1 and 11β-HSD1 positive cells. These indicate that the cadmium reduces the number of Leydig cells possibly before 35 days after EDS. Apparently, loss of Leydig cell numbers account for the reductions in both the serum testosterone levels and the expressions of all these steroidogenic genes/proteins. However, it is still unknown whether the quality of Leydig cells formed (the ability to produce testosterone on per cell base) were also affected by cadmium exposure.

Cadmium exposure may interfere with Leydig cell development by different mechanisms: directly affecting precursor or developing Leydig cell themselves, or indirectly by affecting the micro-environment that regulates Leydig cell development. The evidences suggested strongly that the late most likely play a major, if not exclusive, role. A single low dose of cadmium seemed permanently disrupted non-Leydig cell functions, as indicated by the reduced expressions of FSHR (a Sertoli cell-specific protein) and DHH (a Sertoli-cell and germ cell protein)^25^. The disruption in Sertoli cell functions were further reflected by the elevated expressions of *Gnrhr* and *Fshb* in the pituitary via the negative feed-back mechanism. This is consistent with a previous *in vitro* observation that cadmium was indeed capable of disrupting Sertoli cell function if added directly to the cells^26^. It is well known that Sertoli cells are not only essential for the development of sperms, but also for creating an essential niche for the development of Leydig cells^27^. The significant reduction in DHH may also be contributed by the damages in spermatogenesis, since DHH was also produced by spermatogonia and spermatocytes^25^. Studies have demonstrated that Sertoli cell is the most important cell that regulates Leydig cell development and steroidogenesis, by secreting regulatory factors, such as DHH^28^. The factor that plays a major role in Leydig cell development could be DHH. DHH is expressed by Sertoli cells^29^, spermatogonia and spermatocytes^25^. DHH has been shown to be one of the most important regulatory factors in the early stage of Leydig cell development. DHH binds to its receptor Patched 1^30^ to trigger Leydig cell differentiation by up-regulating steroidogenic factor 1 and *Cyp11a1* expression^30^. Mutation of DHH in mice not only disrupted the formation of fetal Leydig cells^31^ but also blocked the formation of adult Leydig cell population^28, 32^. The reduction in DHH in the present study strongly suggests that cadmium is capable of affecting the regulatory environment that is necessary for the development of Leydig cells. Indeed, using an *in vitro* stem Leydig cell differentiation model, we demonstrated that DHH and LH worked together to induce the differentiation of Leydig cells from the stem cells.

In addition to the local environment, cadmium treatment also affected the factors from the distance. Interestingly, factors from the pituitary were not affected equally. Unlike *Gnrhr and Fshb*, which were significantly elevated via negative feedback mechanism after the disruption of Sertoli cell functions, *Lhb* expression level was significantly reduced, leading to the reduced serum LH levels (Fig. 1B). Normally, serum LH levels were elevated after EDS treatment^23, 24^. The failure to elevate LH or to its control levels, in the face of lower testosterone level and lower expression levels of Leydig cell specific genes, suggesting strongly that cadmium can directly target pituitary to inhibit expression of *Lhb*. This effect appears to be very specific since the expressions of other two pituitary genes *Gnrhr* and *Fshb* went to the opposite direction. Study is currently underway in our lab to elucidate this specific effect. In addition, since cadmium can stay in the body for a long period of time, it could be important to establish how long the effect of single dose of cadmium lasts for in the development of Leydig cells in future studies. Therefore, a brief exposure to cadmium also permanently disrupts *Lhb* expression in pituitary, thus thereafter affecting Leydig cell regeneration. Indeed, DHH and LH worked together to induce the differentiation of stem Leydig cells, although either of them is ineffective.

In conclusion, a brief exposure to low dose of cadmium can significantly affect Leydig cell development in adult animals. The effect seems most like through disrupting regulatory factors both locally (DHH by Sertoli and germ cells) and in distance (LH by pituitary). Both factors are critical in the development of Leydig cells, with DHH necessary for the commitment of stem cells into the Leydig lineage and LH for the maturation of the cells along the lineage. Future works are needed to examine whether cadmium is capable of affect Leydig cell developments in the fetal and pubertal periods.

## Electronic supplementary material

Supplementary Information
