# Supplementary material for: A brief exposure to cadmium impairs Leydig cell regeneration in the adult rat testis
Source: Sci Rep. 2017 Jul 24;7:6337. doi: 10.1038/s41598-017-06870-0 (PMC5524795; doi:10.1038/s41598-017-06870-0)

**A brief exposure to cadmium impairs Leydig cell regeneration in the adult rat testis**

Xiaolong Wu ^1a^, Xiaoling Guo ^2a^, Huang Wang ^2^, Songyi Zhou ^1^, Lili Li ^1^, Xiaomin Chen ^1^, Guimin Wang^1^, Jianpeng Liu ^2^, Hong-Shan Ge ^2,**^, Ren-Shan Ge ^1,2,*^

^1^ Department of Anesthesiology and ^2^ Center of Scientific Research, The Second Affiliated Hospital and Yuying Children’s Hospital, Wenzhou Medical University, 109 Xueyuan West Road, Wenzhou, Zhejiang 325027, China

^a^ These authors contributed equally to this work.

Running title: Cadmium impairs Leydig cell regeneration

** Co-Correspondence: Hongshan Ge, Ph.D., Center of Scientific Research, The Second Affiliated Hospital & Yuying Children's Hospital of Wenzhou Medical University, Wenzhou, Zhejiang 325027, China, dafeng76@126.com

* Correspondence: Ren-Shan Ge, M.D., Department of Anesthesiology, The Second Affiliated Hospital & Yuying Children's Hospital of Wenzhou Medical University, Wenzhou, Zhejiang 325027, China, Tel: 001-86-1390-577-7099, r_ge@yahoo.com (RS Ge)

**Supplementary table 1. Antibodies**

| **Antibody** | **Species** | **Vendor (City, State, catalogue)** | **Dilution** | |
| --- | --- | --- | --- | --- |
|  |  |  | **WB** | **HS** |
| β-actin | rabbit | Cell Signaling Technology (Danvers,  MA,12620) | 1:1000 | ND |
| LHCGR | goat | Santa Cruz (Santa Cruz, CA,  sc-26343) | 1:1000 | ND |
| STAR | rabbit | Abcam (San Francisco, CA,  ab133657) | 1:1000 | ND |
| CYP11A1 | rabbit | Santa Cruz (Santa Cruz, CA,  sc-18043) | 1:1000 | ND |
| 3β-HSD1 | rabbit | Abcam (San Francisco, CA,ab65156) | 1:2000 | 1:1000 |
| CYP17A1 | rabbit | Santa Cruz (Santa Cruz, CA,  [sc-66850](https://www.scbt.com/scbt/product/cyp17a1-antibody-m-80?requestFrom=search)) | 1:1000 | ND |
| 11β-HSD1 | rabbit | Abcam (San Francisco, CA,  ab39364) | 1:2000 | 1:1000 |
| FSHR | rabbit | Abcam (San Francisco, CA,ab103874) | 1:2000 | ND |
| DHH | rabbit | Proteintech (Rosemont, IL,  13889-1-AP) | 1:2000 | ND |

ND = Not detected; WB = Western blot; HS = Histochemical staining.

**Supplementary table 2**. Primer information

| **Primer**  **Symbol** | **Gene name** | **Primer direction** | **Sequences (5’to 3’)** | **PCR**  **(bp)** | **Accession** |
| --- | --- | --- | --- | --- | --- |
| Lhcgr | Luteinizing hormone receptor | Forward | CTGCGCTGTCCTGGCC | 103 | NM_012978 |
|  |  | Reverse | CGACCTCATTAAGTCCCCTGAA |  |  |
| Scarb1 | Scavenger receptor class B, member 1 | Forward | ATGGTACTGCCGGGCAGAT | 117 | NM_031541 |
|  |  | Reverse | CGAACACCCTTGATTCCTGGTA |  |  |
| Star | Steroidogenic acute regulatory protein | Forward | CCCAAATGTCAAGGAAATCA | 187 | NM_031558 |
|  |  | Reverse | AGGCATCTCCCCAAAGTG |  |  |
| Cyp11a1 | Cholesterol side chain cleavage enzyme | Forward | AAGTATCCGTGATGTGGG | 127 | NM_017286 |
|  |  | Reverse | TCATACAGTGTCGCCTTTTCT |  |  |
| Hsd3b1 | 3β-Hydroxysteroid dehydrogenase 1 | Forward | CCCTGCTCTACTGGCTTGC | 189 | NM_001007719 |
|  |  | Reverse | TCTGCTTGGCTTCCTCCC |  |  |
| Cyp17a1 | P450 17α-hydroxylase/ 17,20-lyase | Forward | TGGCTTTCCTGGTGCACAATC | 90 | NM_012753 |
|  |  | Reverse | TGAAAGTTGGTGTTCGGCTGAAG |  |  |
| Hsd17b3 | 17β-Hydroxysteroid dehydrogenase 3 | Forward | TGAAAGTTGGTGTTCGGCTGAAG | 202 | NM_054007 |
|  |  | Reverse | TGAAAGTTGGTGTTCGGCTGAAG |  |  |
| Hsd11b1 | [11β-Hydroxysteroid dehydrogenase 1](https://www.baidu.com/link?url=RASn5FVJOHQO5F8yLZuLK2GaE-txBvaDG-Aix0zS1TOK-H6BDM3SQ-dtmZqJCiib&wd=&eqid=adb6e42d00037867000000045854b8a0) | Forward | GAAGAAGCATGGAGGTCA | 290 | NM_017080 |
|  |  | Reverse | CTCAAGATTATCCCAGAGG |  |  |
| Amh | Antimulerian hormone | Forward | GCCCTAACCCTTCAACCA | 82 | NM_012902 |
|  |  | Reverse | GGGAATCAGAGCCAAACAGA |  |  |
| Fshr | Follicle stimulating hormone receptor | Forward | CCACAAGCCAATACAAACTAACT | 327 | NM_199237 |
|  |  | Reverse | CAAAAGTCCAGCCCAATACC |  |  |
| Dhh | Desert Hedgehog | Forward | AACCCCGACATAATCTTCA | 150 | NM_053367 |
|  |  | Reverse | CTCGTCCCAACCTTCAGT |  |  |
| Fshb | Follicle stimulating hormone beta | Forward | TTCACCCACCCTTGTCTT | 135 | NM_001007597 |
|  |  | Reverse | GCTCCTCCTCACTACCTGTC |  |  |
| Gnrhr | Gonadotropin releasing hormone receptor | Forward | cttgaagcccgtccttgg | 441 | NM_031038 |
|  |  | Reverse | gcgatccaggctaatcac |  |  |
| Lhb | [Luteinizing hormone](https://www.baidu.com/link?url=U-Y5YZNki4dcyIGsOTRbXJCYP2nCiCXFLQ6siW0JFh10oJUbvYqeKIyOZZjtDhNWXyK4T0rcNkWaR4C64tFXlWASXKQzhfCpSKeOH1YGbkwC8czaFfXg6ZSmQ1nYvOyU&wd=&eqid=c68c83c00003fa6d000000045854b949) beta | Forward | CTGCTGCTGAGCCCAAGTGT | 127 | NM_012858 |
|  |  | Reverse | TGCTGGTGGTGAAGGTGATG |  |  |
| β-actin | Beta-Actin | Forward | CCATGAAGATCAAGATCAT | 106 | NM_001141945 |
|  |  | Reverse | TTGCTGATCCACATCTGCT |  |  |

The gels of WB are used in the main figures as following:

STAR:


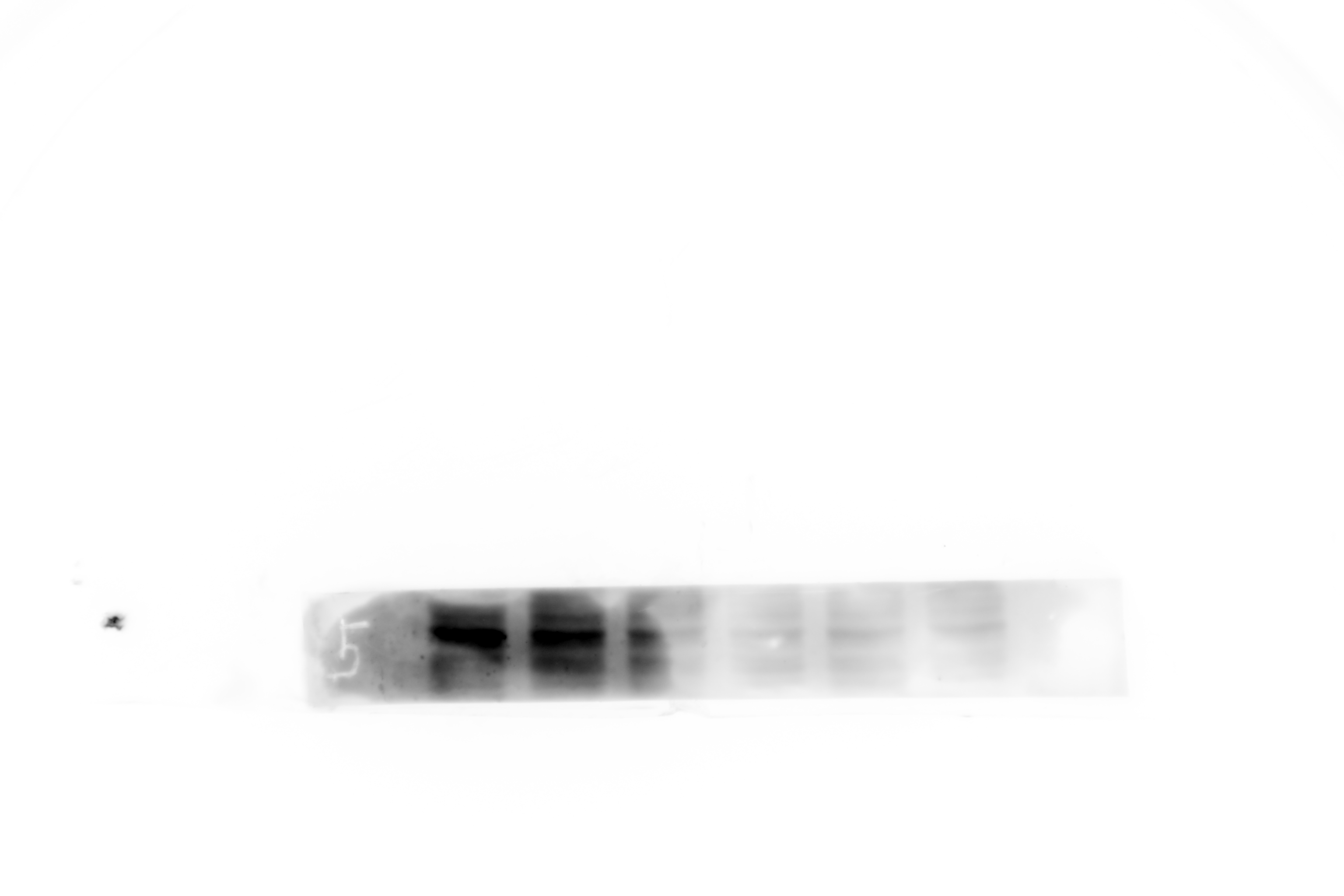


3β-HSD1:


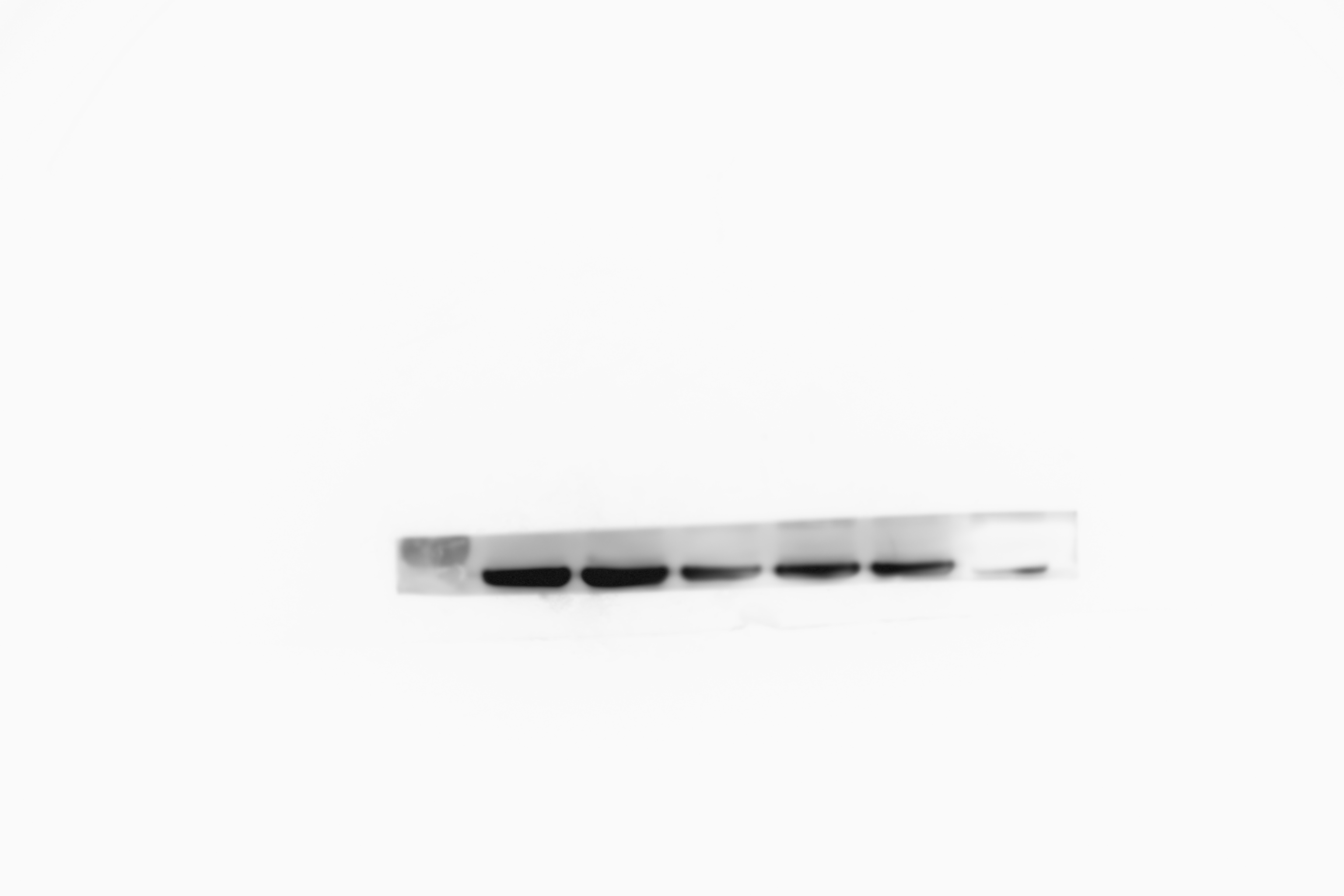


CYP11A1:


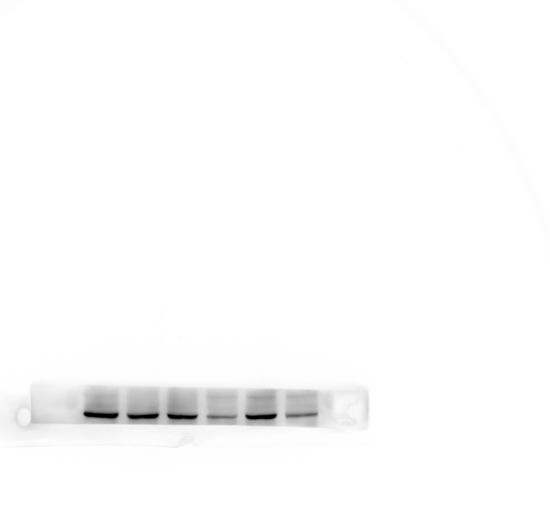


CYP17A1:


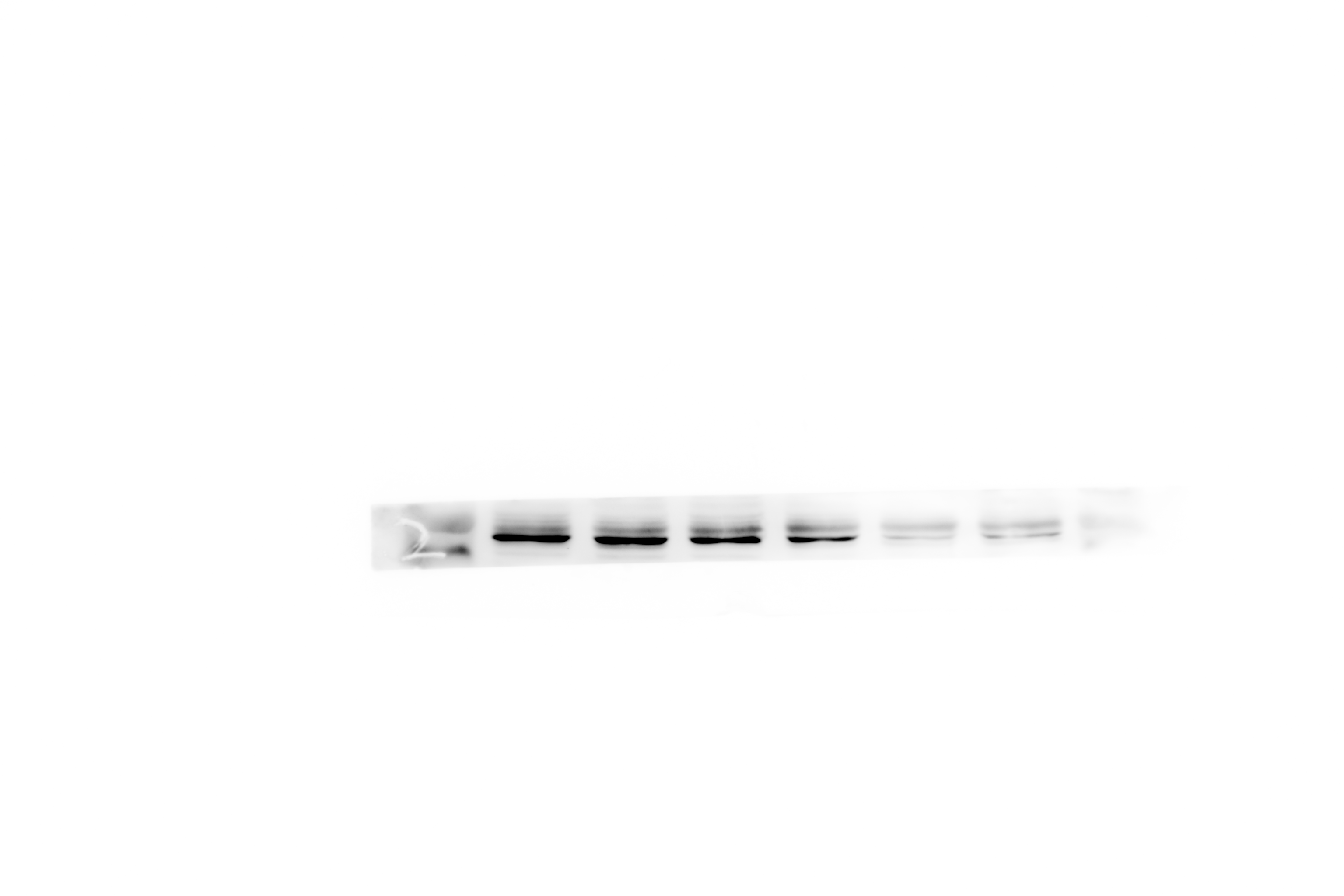


11β-HSD1:


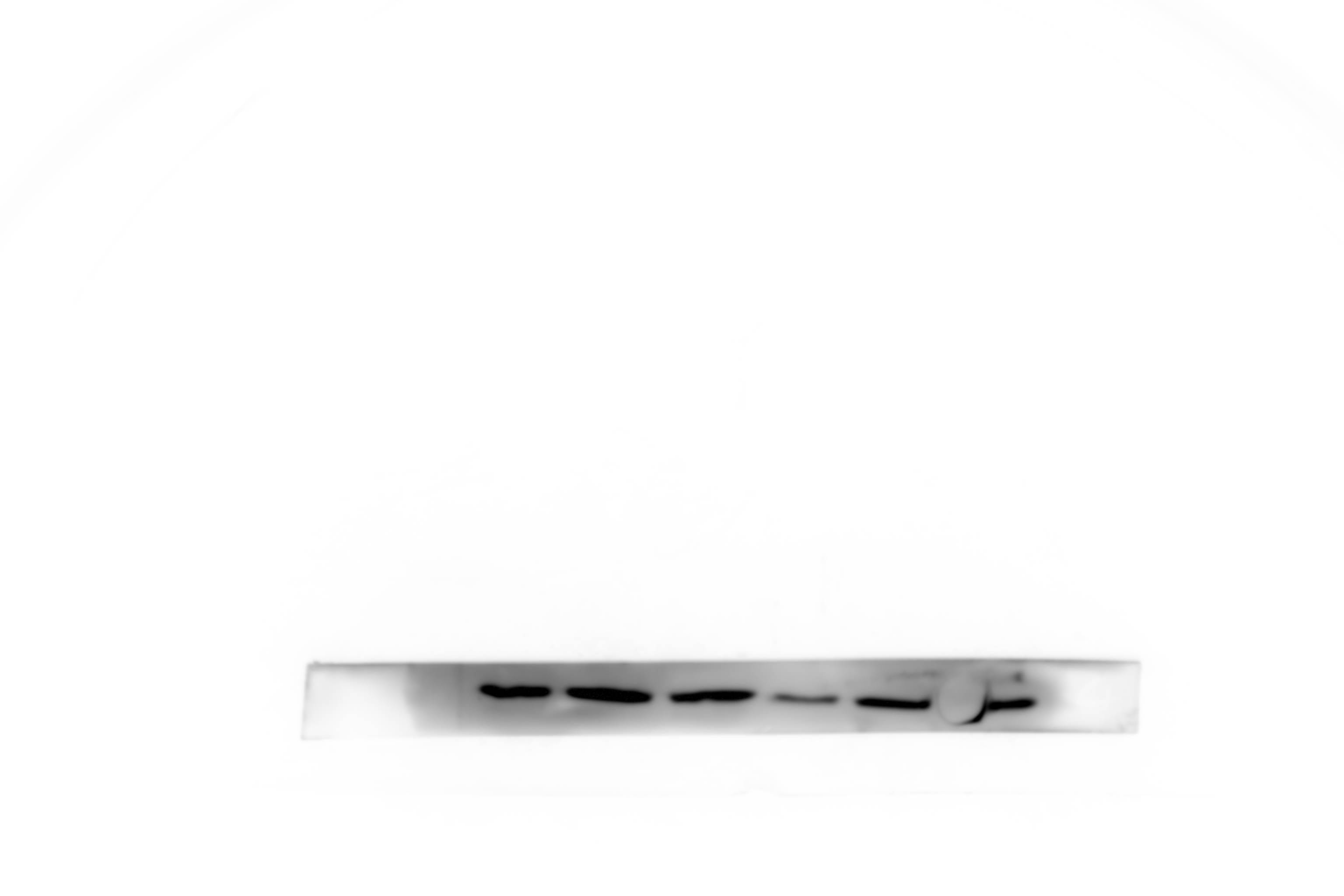


DHH:


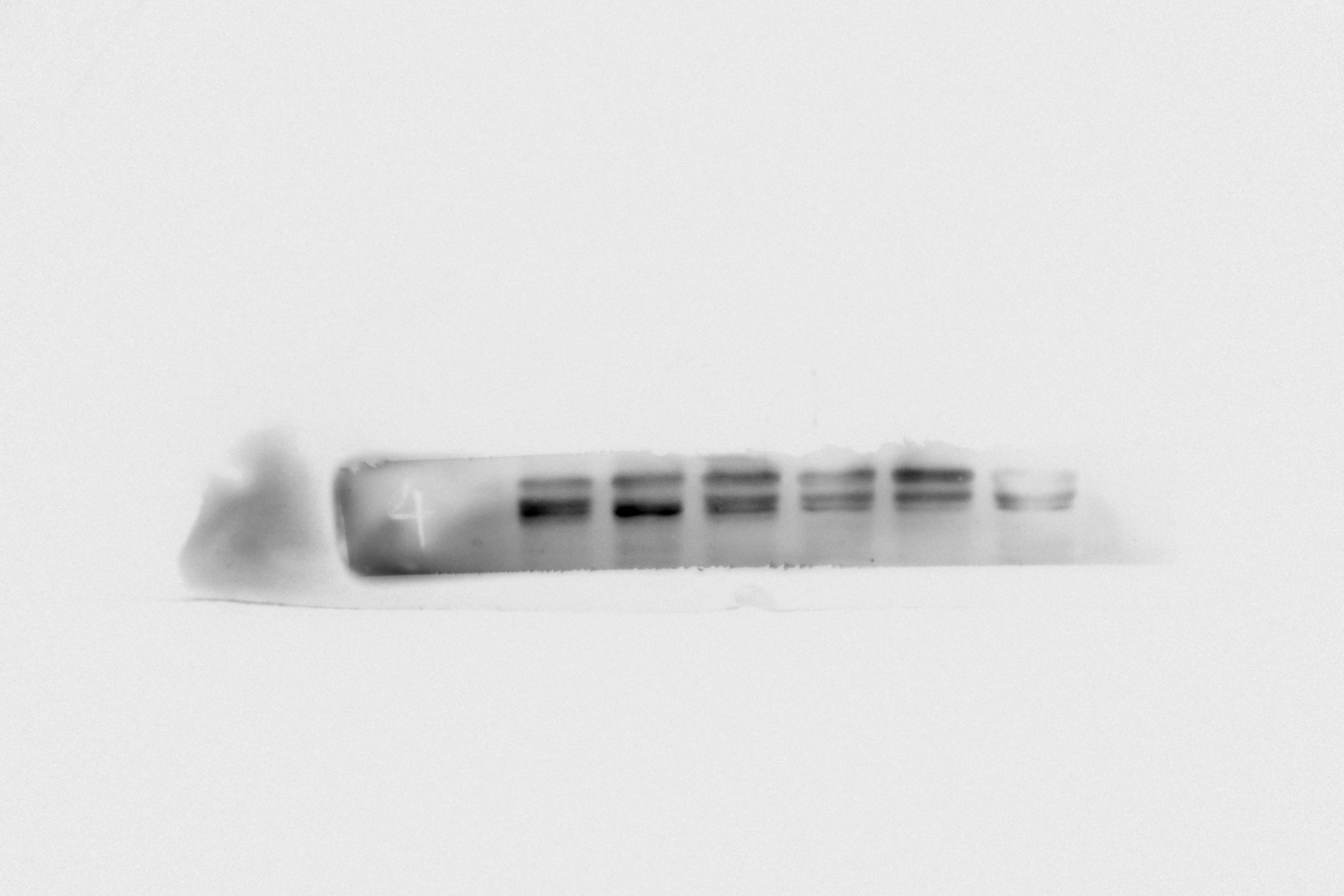


LHCGR:


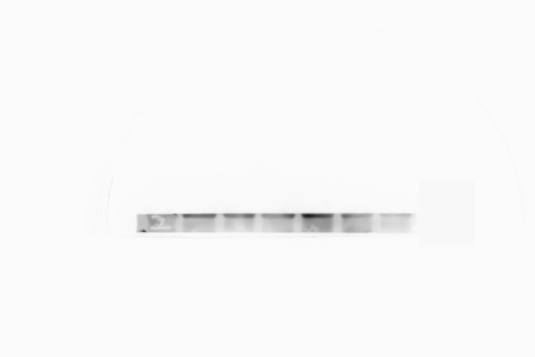


FSHR:


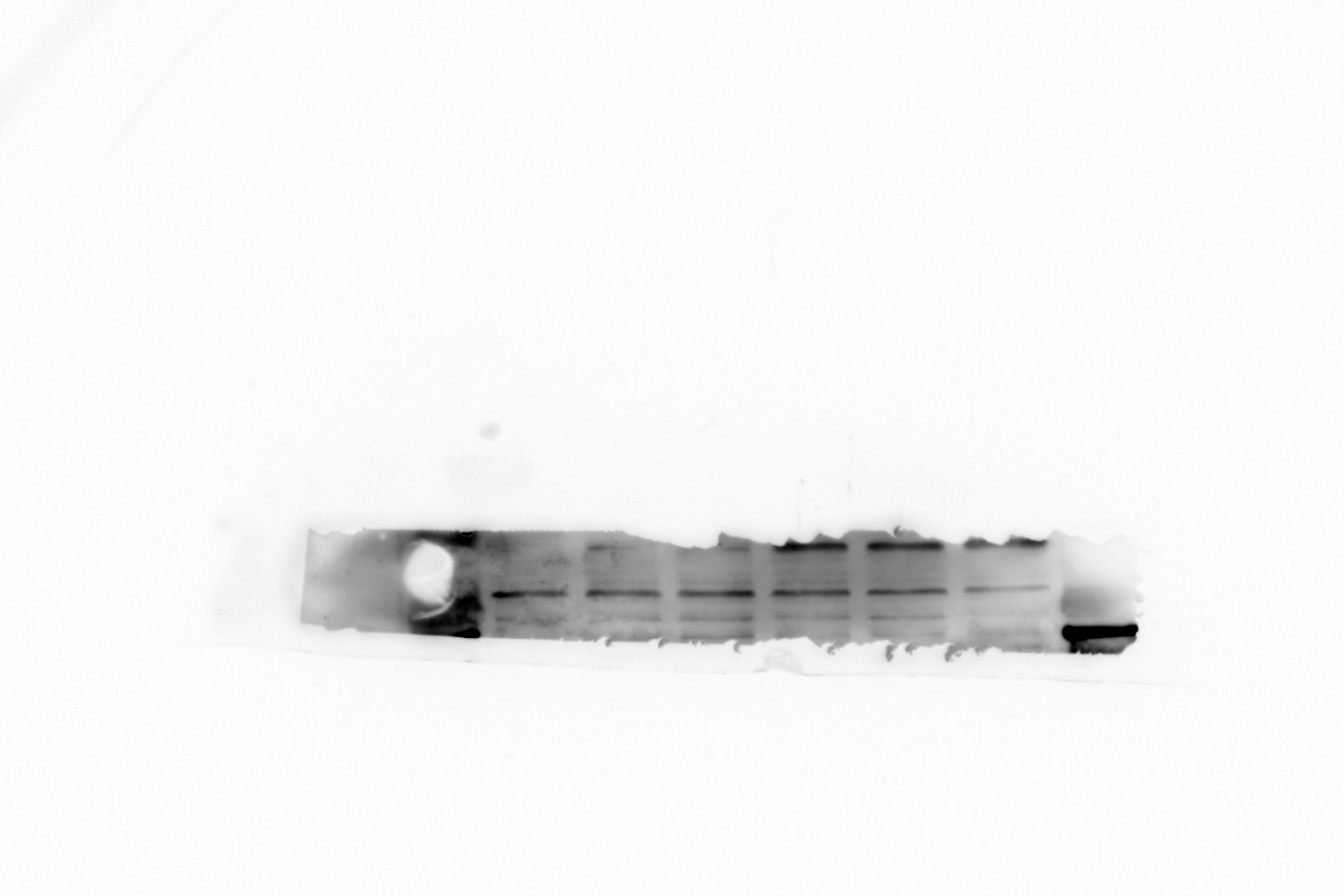


β-actin:


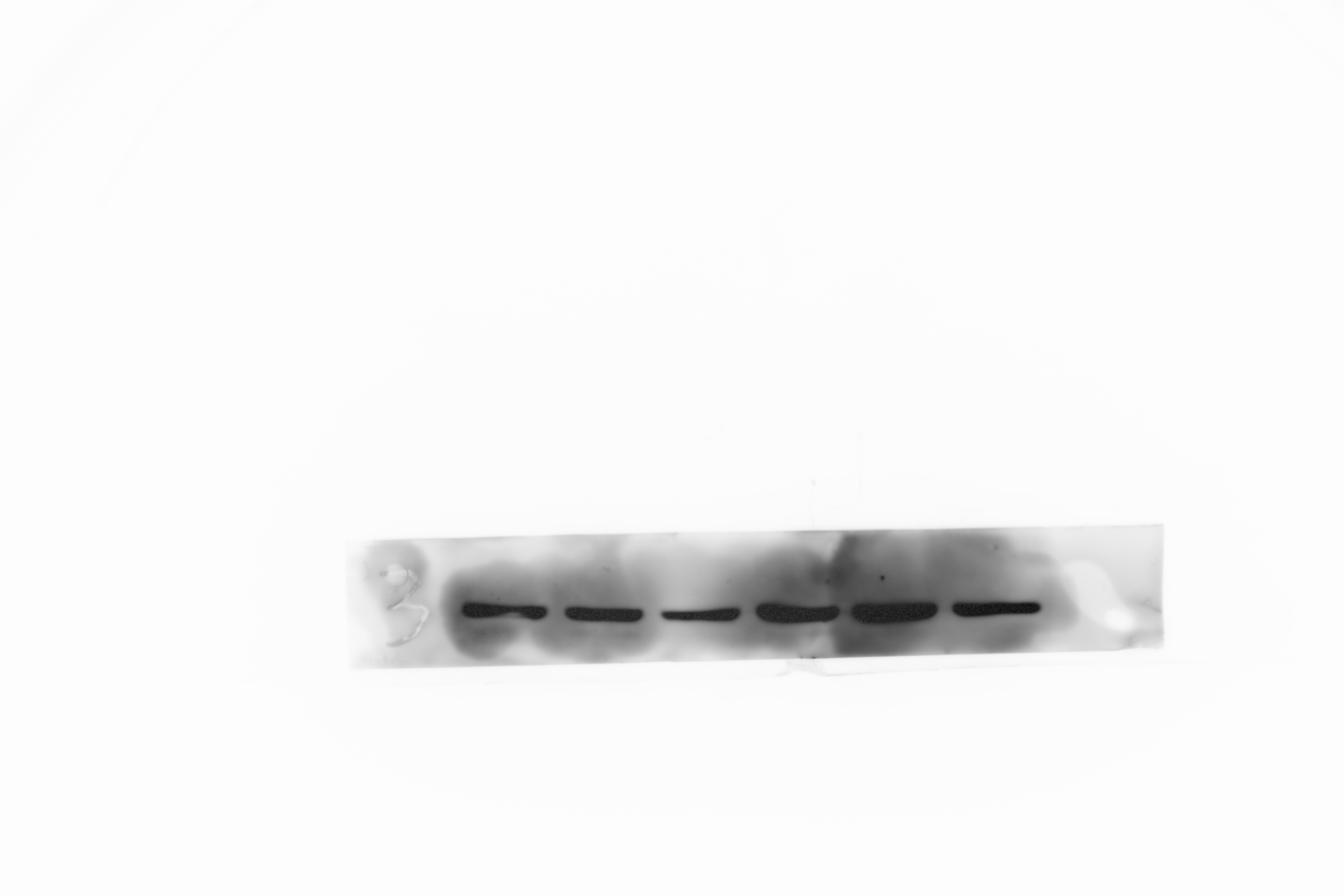

Supplement: Supplementary file 1 — Supplementary Information [file 41598_2017_6870_MOESM1_ESM.doc]
